# Should I stay or should I go? Initiation of joint travel in mother–infant dyads of two chimpanzee communities in the wild

**DOI:** 10.1007/s10071-015-0948-z

**Published:** 2016-02-01

**Authors:** Marlen Fröhlich, Roman M. Wittig, Simone Pika

**Affiliations:** Humboldt Research Group ‘Evolution of Communication’, Max Planck Institute for Ornithology, Eberhard-Gwinner-Straße 9, 82319 Seewiesen, Germany; Department of Primatology, Max Planck Institute for Evolutionary Anthropology, Deutscher Platz 6, 04103 Leipzig, Germany; Taï Chimpanzee Project, Centre Suisse de Recherches Scientifiques, BP 1303, Abidjan 01, Côte d’Ivoire

**Keywords:** Communication, Gestures, Acquisition, Social negotiation, Chimpanzee, *Pan troglodytes verus*, *Pan troglodytes schweinfurthii*

## Abstract

It is well established that great apes communicate via intentionally produced, elaborate and flexible gestural means. Yet relatively little is known about the most fundamental steps into this communicative endeavour—communicative exchanges of mother–infant dyads and gestural acquisition; perhaps because the majority of studies concerned captive groups and single communities in the wild only. Here, we report the first systematic, quantitative comparison of communicative interactions of mother–infant dyads in two communities of wild chimpanzees by focusing on a single communicative function: initiation of carries for joint travel. Over 156 days of observation, we recorded 442 actions, 599 cases of intentional gesture production, 51 multi-modal combinations and 80 vocalisations in the *Kanyawara* community, Kibale National Park, Uganda, and the *Taï* South community, Taï National Park, Côte d’Ivoire. Our results showed that (1) mothers and infants differed concerning the signal frequency and modality employed to initiate joint travel, (2) concordance rates of mothers’ gestural production were relatively low within but also between communities, (3) infant communicative development is characterised by a shift from mainly vocal to gestural means, and (4) chimpanzee mothers adjusted their signals to the communicative level of their infants. Since neither genetic channelling nor ontogenetic ritualization explains our results satisfactorily, we propose a revised theory of gestural acquisition, *social negotiation*, in which gestures are the output of social shaping, shared understanding and mutual construction in real time by both interactants.

**Electronic supplementary material:**

The online version of this article (doi:10.1007/s10071-015-0948-z) contains supplementary material, which is available to authorized users.

## Introduction

Across cultures and languages, human children enter language hands first. It has been hypothesised that this brief period in human ontogeny recapitulates phylogeny, with gestures being the modality out of which human language may have blossomed (for an overview, see Hewes 1973). This so-called gesture-first hypothesis especially inspired comparative researchers to search for evolutionary precursors to human language in non-human primate gesturing (Tomasello 2008). Systematic studies in the last decades have shown that gestures indeed are used as intentionally produced, elaborate and flexible communicative strategies and play, similar to vocalisations, a crucial role in great apes’ everyday communication (for overviews, see Call and Tomasello 2007; Pika and Liebal 2012). While there is a large body of work focusing on the description of gestural repertoires in a variety of different primate species (Call and Tomasello 2007; Genty et al. 2009; Hobaiter and Byrne 2011), usage of distinct gesture types (Leavens et al. 1996; Pika and Mitani 2006) and cognitive mechanisms underlying gestural signalling (Genty and Zuberbühler 2014; Liebal et al. 2004; Pika and Mitani 2006; Roberts et al. 2014b), surprisingly little is known about the first step into this communicative endeavour: mother–infant coordination as co-regulated social interaction (King 2004).

A large body of research has been emphasising the benefit of conceptualising the mother–infant dyad as a system decades ago (for a review, see van de Rijt-Plooij and Plooij 1987). This system assumes that the mother–infant dyad behaves as an organised whole characterised by mutual modification of each other’s behaviour in response to feedback (Watzlawick et al. 1967). Pioneering work has been carried out by Plooij (1978, 1979) 40 years ago, who investigated gestural ontogeny in mother–infant communication in chimpanzees at Gombe, Tanzania. He showed that, similar to communicative development in human children, interactions between chimpanzee infants and their mothers slowly progress, with a shift around the ages of 9–12 months from acts without social–communicatory intention to intentional acts. At this age, the infant is able not only to *maintain* an interaction, e.g. ‘play-tickling’, but also to *initiate* it by using behaviours whose values have been established in earlier sessions (Plooij 1978). Plooij thus concluded that gestures in chimpanzees do not represent innate signals but are acquired through a process of ‘social negotiation’ (also termed ‘conventionalisation’; Mead 1910). This idea was later developed into a formal hypothesis, ‘ontogenetic ritualization’ (OR), in which the forms that gestures take derive directly from repeated social interactions in which individuals participate (Tomasello et al. 1994). Thus, evidence for the process of OR would be high degrees of individual variation within dyads, groups and between communities but also concerning the means used to achieve the same goals. Halina and colleagues (2013) recently investigated mother–infant coordination for the purpose of joint travel (carries) in captive bonobo (*Pan paniscus*) mother–infant dyads and were able to attribute the process of OR to several carry-initiating gestures. This study, thus, supported the hypothesis of Tomasello and colleagues (1994; Call and Tomasello 2007) that gestures are acquired via repeated social interactions. For current purposes, the term individual learning refers to a process in which two or more individuals independently acquire the same behaviour due to ‘similar learning environments’ (Whiten and Ham 1992). Contrarily, the term social learning is used to indicate situations in which individuals learn distinct behaviours by imitating (Bandura 1986) but also by interacting and observing each other. Recently, Byrne and colleagues (Genty et al. 2009; Hobaiter and Byrne 2011) challenged the idea that learning plays a role in great ape’s gestural production and suggested that similarly to vocal production and facial expressions, gestures appear hard-wired and can be explained as a result of genetic channelling during development alone. This hypothesis is in contrast to great apes’ high degree of manual flexibility in other behavioural domains such as food processing and tool use, and considerable inter-site variability (Byrne et al. 2011; van Schaik et al. 2003; Whiten et al. 1999). However, since systematic quantitative comparisons of gestural signalling in wild populations are still lacking, the absence of evidence might merely reflect a paucity of data, rather than a lack of gestural complexity on behalf of the apes.

The aim of the present study was to gain a better understanding of the complexity and variability of communicative exchanges of mother–infant dyads and to shed light on gestural acquisition. To do so, we enabled the first systematic quantitative comparison of gestural signalling in two chimpanzee communities of different subspecies in their natural environments (*Kanyawara*, Kibale National Park, Uganda, and *Taï South*, Taï National Park, Côte d’Ivoire). Since other studies (Halina et al. 2013; Plooij 1978) had suggested that the communicatory context of joint travel represents a promising candidate for frequent communicative exchanges between mother–infant dyads about a distinct goal (leaving a location), we focused our research efforts on this single communicative function. To enable horizontal comparisons between individuals of different communities *and* vertical comparisons of the same individuals, behavioural data were collected in two consecutive years. This important methodological tool for understanding the cognitive prerequisites underlying different communicative skills had so far only been employed in captive settings (Pika et al. 2003; Schneider et al. 2011; Tomasello et al. 1997).

We addressed the following three questions:

First, which behaviours do chimpanzees employ to initiate joint travel? Plooij (1978), for instance, had noted that mothers who initiate joint travel (1) lower their bottoms, (2) look back at their infants, (3) reach back towards him/her and (4) make tonal grunts. They thus employ a complex set of gestures (lowerback, lookback, reachback^1^) and multi-modal combinations (lookback and grunt) to communicate the distinct goal of joint travel and also the direction to travel to. To investigate this question, we compiled individual repertoires of behaviours produced to initiate joint travel and analysed signal production in terms of gesture category (e.g. visual or tactile) and signal modality (gesture, vocalisation or combinations of the two, i.e. multi-modal signals). We expected chimpanzee mothers in the wild to be the main carry initiators, thereby contributing the majority of travel-initiating behaviours (van Lawick-Goodall 1967).

Second, are gesture types employed to initiate joint travel due to learning (including both individual and social learning) between mothers and infants or can their production simply be explained as a result of genetic channelling? Since it is impossible to observe developmental processes as they unfold over time under natural conditions, a window approach onto gesture acquisition was applied: We investigated the degree of variability in gestural production to initiate joint travel within dyads within communities and between communities (Pika et al. 2003, 2005). Furthermore, since the presence of idiosyncratic gestures is a key indicator of individual learning and evidence against a phylogenetic origin of gestures, we examined whether idiosyncratic gestures were employed (found to be used by only a single individual of the whole community over two subsequent years and study periods). Pronounced variability in individual gestural production across dyads and communities (e.g. low concordance rates between individuals’ repertoires and idiosyncratic gestures) would provide evidence for the impact of learning in mother–infant communication, whereas high rates of concordances in gestural variability across dyads and communities may imply genetic channelling.

Third, do chimpanzee mothers adjust their behaviour to the developmental stage of their infants, and how does infant age influence signal production in both mothers and infants? As suggested by Plooij (1978), the means mothers employ to communicate with their infants might be influenced by the developmental shift from actions to intentional communication in young chimpanzees. In addition, a mother’s accumulated experience in interactions with previous offspring might also shape the carry interaction substantially, as well as the prevailing behavioural context (i.e. varying necessity to carry). For instance, while frequent gestural interactions can often be observed in evolutionarily non-urgent, or ‘relaxed’, situations (e.g. playing and grooming; Pika 2014; van Lawick-Goodall 1967), they sometimes outrival vocalisations in evolutionary ‘urgent’ contexts, where silent communication transfer is an advantage (e.g. consortship; Hobaiter and Byrne 2012).

## Methods

### Study sites and subjects

The study investigated the communicative behaviour of  mother–infant dyads in two different chimpanzee communities: *Kanyawara* in Kibale National Park, Uganda (*Pan troglodytes schweinfurthii*), and *Taï South* in Taï National Park, Côte d’Ivoire (*P. t. verus*). Detailed descriptions of the study areas can be found in Wrangham and colleagues (1992) and Boesch and Boesch-Achermann (2000). During the two study periods, the size of the *Kanyawara* group varied between 53 and 56 individuals, respectively, 21 and 24 in *Taï South*. The *Kanyawara* and *Taï* chimpanzees are well habituated and have been studied regularly since 1987 (Wrangham et al. 1992) and 1979 (Boesch and Boesch-Achermann 2000), respectively, enabling dawn-till-dusk follows and the collection of high-quality recordings. In addition, we had access to long-term data concerning the chimpanzees’ demography, social relationships, relatedness and ranks. We observed communicative interactions of a total of 13 mother–infant dyads (seven from *Kanyawara* and six from *Taï South*), with offspring ranging from 9 to 69 months of age (see Table 1). At *Taï* one mother gave birth to another infant in the second field period; hence, we observed 12 chimpanzee mothers and 13 infants.

**Table 1:** Information on observed mother–infant dyads with respective observation time and raw data set

Study site	Dyad (infant/mother)	Infant sex	Infant age P1 (months)	Infant age P2 (months)	Observation time (h)	Video-recorded interactions (h)
Kanyawara	Winza/Wangari	M	9–11	21–23	105	15.2
	Tembo/Tenkere	M	13–15	25–27	119	18.4
	Mango/Michelle	F	13–15	25–27	87	7.3
	Lily/Leona	F	3–5^a^	15–17	60	7.2
	Thatcher/Tongo	F	16–18	28–30	112	15
	Gola/Outamba	F	48–50^b^	N/A	45	7.2
	Wallace/Wilma	M	55–57	67–69	73	10.1
Taï South	Mohan/Mbele	F	10–12	22–24	150	11.2
	Iniesta/Isha	M	N/A	10–12	91	12
	Solibra/Sumatra	M	15–17	27–29	147	14.7
	Jeff/Julia	M	15^c^	N/A	20	0.4
	Kayo/Kinshasa	F	19–21	31–33	148	17.0
	Ithaka/Isha	M	64–66^b^	N/A	41	9.5
Σ	13	6:7	11	10	1198	145.2

The last line provides the total sample size for each column (P1/P2: first/second period of data collection)

^a^P1 not included since infant was too young

^b^Mothers gave birth to sibling in P2, thus no P2 data available

^c^Deceased on November 1, 2012

### Data collection

Observations were made on chimpanzees of the *Kanyawara* community in Kibale National Park and the *Taï South* group at Taï National Park during four periods between October 2012 and June 2014 (*Kanyawara*: March–May 2013, March–June 2014; *Taï South*: October–December 2012, October–December 2013). We used a focal behaviour sampling approach (Altmann 1974), while maintaining a record of the frequency with which a particular dyad had been observed. In situations where we could choose which of several dyads to film, we targeted those individuals previously sampled least often. Following Hobaiter and Byrne (2011), who had suggested that approximately 15 h of active gesture time or approximately 150 days of field observation time would enable to assess the *whole* gestural repertoire of a given chimpanzee community (*N* = 82), we observed all 13 mother–infant dyads for a total of 156 days. All social interactions of mothers and infants (i.e. mother–infant interactions as well as mother-conspecific and infant-conspecific interactions) that were judged to have any potential for communicative interactions were recorded using a digital high-definition camera (Canon HF M41) with an external unidirectional microphone (Sennheiser K6). This method resulted in a total of 169 h of video footage recorded during approximately 1198 h of focal observations (see Table 1 for further details). However, the present paper focuses only on the communicative context of carry initiation; thus, our analysis is based on a total of 410 high-quality recordings of mother–infant behaviour in this respective context (mean recordings per dyad: 33.2). In addition, every 15 min we conducted a focal scan by using a Personal Digital Assistant (HP iPAQ rx1959) with focal/time sampling utilised as sampling/recording rule (Altmann 1974). This method enabled us to collect data on a variety of additional parameters such as behavioural context and party composition (see Online Resource 1, Table S2), resulting in a total of 4505 behavioural scans.

### Video coding procedure

To establish the behavioural repertoires of mothers and infants used to initiate maternal carries and enable subsequent analyses, a total of 410 high-quality video files of mother–offspring carry initiations (i.e. carries with clear visibility of carry-initiating behaviours) were coded using the program Adobe Premiere Pro CS4 (version 4.2.1.). In addition, we included PDA recordings of five interactions, resulting in a total of 415 interactions. Behavioural definitions were based on established ethograms of the behaviour of two long-term studies of eastern chimpanzees (Goodall 1986; Nishida et al. 1999) and several gesture studies (Call and Tomasello 2007; Hobaiter and Byrne 2011; Roberts et al. 2014a). Based on parameters used in previous work on great ape gesturing (Pika et al. 2003, 2005; Pika and Mitani 2006), a coding scheme was developed. For our purposes, all analysed joint travel events included maternal carries (i.e. involving mother–infant body contact). While coding all agent-initiated carries, we differentiated between carry-initiating behaviours via (1) physical actions, (2) intentionally produced gestures, (3) multi-modal combinations (gesture plus vocalisation) and (4) vocalisations. A physical action was defined as any behaviour that resulted in joint travel through direct manipulation of another’s body or the movement of one’s own body into a carry position. Carry-initiating actions included, for instance, grabbing, forcibly pulling, lifting or approaching another individual (see Online Resource 1, Table S1). Gestures were defined as directed, mechanically ineffective movements of the body or body postures that elicited (‘requested’) a voluntary response by the recipient (Pika 2008). In addition, we only included those gestures in our analyses that were accompanied by one or more key characteristics of intentional communication (Bates 1976; Bruner 1981; Pika et al. 2003):

*Sensitivity to the attentional state of the recipient* The signaller shows signs of being aware of the recipient’s state of attention, e.g. by using visual gestures only when the recipient is looking.

*Response waiting* The signaller pauses at the end of the signal and waits for at least two second for a response while maintaining visual contact.

*Apparent satisfaction of signaller* The signaller’s communication ceases when the apparent goal has been met by the recipient (Hobaiter and Byrne 2014).

*Goal persistence* The signaller elaborates her signalling when thwarted, e.g. by repeating and exaggerating the signal or by using a different communicative means (Pika et al. 2005; Pika and Mitani 2006).

Gestures were clustered into three signal categories: audible (signals generate a sound while being performed, e.g. slapground), tactile (signals include physical contact with the recipient, e.g. touching) and visual (signals generate a mainly graphic component, e.g. raisearm) signals (Pika et al. 2003). To identify carry initiations, the behaviour of both, the signaller and the recipient throughout the interaction, from first initiating action/gesture to start of carry, was taken into account to assess the success of communicative attempts (Smith 1965). Idiosyncratic gestures, which are exclusive for single individuals in the whole community, had been observed at least three times to be included in the analyses (Pika et al. 2003, 2005). Vocalisations, especially those accompanying gestures (‘multi-modal signals’), were analysed in terms of their broad categories (Crockford and Boesch 2005; Goodall 1986; Table 2). Finally, for each signal or action case, we coded the following parameters: *interaction role of the signaller*: two levels, mother, infant; *infant age*: range 9–69 months; *necessity of carry*: two levels (low; carry preceded by feeding, playing, resting, relaxed group travel; high: preceded by aggressive behaviours such as chasing and hitting, catching-up with already left party/group, displaying and patrolling); *mother’s parity*: number of offspring reared at least until juvenility (plus present infant), range 1–5, *partycomposition*: three levels (mother with dependent offspring only, adult females only, mixed group). A least 15 per cent of all mother–infant interactions were coded for accuracy by a second observer and tested using the Cohen’s kappa coefficient to ensure inter-observer reliability (Altmann 1974). A ‘very good’ level of agreement was found for gesture type (*κ* = 0.878), signal type (*κ* = 0.811), signal category (*κ* = 0.843) and necessity of carry (*κ* = 0.816). The level of agreement for carry initiator (mother/infant) was ‘good’ (*κ* = 0.799).

**Table 2:** Gesture and vocalisation types produced to initiate carries in chimpanzee mother–infant dyads identified in this and other studies on wild groups in Budongo (Hobaiter and Byrne 2011; Roberts et al. 2014a); Gombe (Goodall 1986) and Mahale (Nishida et al. 1999)

Gesture/vocalisation	Definition (this study)	Used by	Budongo	Gombe	Mahale
*Audible*					
Loud scratch [ls]	Signaller makes deliberate scratching movements on own body	Mother	Big loud scratch	Self-scratch	Scratch self signalling
Slap ground [sg]	Signaller hits ground with flat palm of his hand	Mother	Slap object	Slap ground	Slap branch
*Tactile*					
Arm on [ao]	Signaller places palm on recipient’s back (>2 s)	Infant	Hand on	–	–
Shake back* [sb]	Signaller shakes lower back in an upward movement when recipient is already clinging	Mother	–	–	–
Scoop infant [rb]	Signaller reaches behind himself and gently pushes infant up onto back with a back ward and upward movement	Mother	Scoop	Scoop	Scoop infant
Touch [to]	Signaller makes short (>2 s) contact with recipient using palm and/or fingers	Both	Touch inner hand	Touch	Touch
Directed push [pu]	Signaller uses limbs or body to bring recipient in direction of movement	Mother	Directed push	Pull towards hand leading	Push ahead
Pull [pl]	Signaller moves recipient’s body part towards himself	Both	Pull	Pull	Pull
put ventral [pv]	Signaller pushes recipient in ventral region	Mother	–	–	Put ventral
*Visual*					
Backward sweep [bs]	Signaller stretches arm towards behind himself in a short, rapid movement	Mother	Backward sweep	Climb aboard	–
Extend leg [el]	Signaller extends leg to facilitate climb onto self	Mother	Present leg	–	Extend leg as ladder
Look [lo]	Signaller gazes at recipient (>2 s)	Both	Look	Wait	Look; wait
Lower back [lr]	Signaller, in lateral position to recipient, lowers abdomen without stopping locomotion	Mother			
Stop and look back/down [lb/ld]	Signaller stops with body orientated in direction of movement and looks back (or down) at recipient	Both	–	–	Look back
Present back/shoulder/venter [pb, ps, pv]	Signaller offers back/venter to recipient	Mother	Present	Present	Present
Reach [ra]	Signaller extends arm towards recipient	Both	Reach	Extend hand	Extend hand
Rear up* [ru]	Signaller briefly rises straight up on two feet while positioned towards recipient	Mother	–	–	–
Turn Bipedal* [tb|	Signaller turns towards recipient with short bipedal movement	Mother	–	–	–
*Vocalisations*					
Hoo whimper [hoo]	Signaller utters a series of soft, low pitch sounds that may become progressively louder and higher in pitch	Infant	–	Whimper	Whimper
Soft grunt [sgr]	Signaller utters a soft, barely voiced sound	Mother	–	Soft grunt	Grunt

### Statistical analyses

Since Byrne and colleagues (Genty et al. 2009; Hobaiter and Byrne 2011) had argued that differences in gestural repertoires of captive apes were simply premature assumptions, with repertoires yet to reach asymptote, we plotted the cumulative numbers of observed gesture types over time for all individuals. If an asymptote was reached (i.e. no further gesture types were observed), we concluded that we had observed the individual’s full repertoire for the specific communicative function of maternal carries. We measured the relationship between an individual’s final repertoire size and the total time that individual had been observed using the Spearman R statistic. For our repertoire analyses, we included only individuals observed for over 60 h (*N* = 10; observation time range 60.25–150 h, mean ± SD = 109.3 ± 32.1 h), which have reached the critical asymptote, to make sure that the complete repertoire of these individuals was grasped within the observation time. We compared repertoire sizes of mother and infants using an independent-samples *t* test after the underlying assumptions were tested (Levene’s test for equality of variances).

To enable a better understanding of gestural acquisition, the gestural repertoires of mothers of the two communities of *Kanyawara* and *Taï South* were compared. To assess concordance rates of gestural repertoires within dyads, within groups and between groups, we used the Dice coefficient (D_*c*_), which ranges from 0 to 1 (Dice 1945). A value of 0 means that two individuals have no gesture types in common, while a value of 1 would mean that the two gestural repertoires are identical. Since chimpanzee infants had very limited gestural repertoires in the specific context of carry initiation, we restricted this particular analysis to maternal repertoires only. In addition, we included in the analysis only data of individuals, whose repertoires had reached asymptote. To investigate whether repertoire similarity was larger between mothers of the same community than between mothers of different communities, we used a matrix permutation test (Sokal and Rohlf 1995).

To test to which extent the predictor variables such as infant age, interaction role, carry necessity and mother’s parity influenced signal type (action, visual gesture, tactile gesture; response variables), we used generalised linear mixed models (GLMM; Baayen 2008) with a binomial error structure and logit link function. We fitted one model for each of the three response variables. Into this, we included interaction role, infant age, carry necessity and mother’s parity as our key test predictors, respectively. Another model was specified for carry initiator as binomial response variable (0 = mother initiation, 1 = infant initiation), but only infant age and parity were specified as key test predictors in this model. Since the average age varied considerably between infants but also within them, we used the method of within-subject centring (van de Pol and Wright 2009). This method allows to test whether the effect of age takes place largely across subjects (cross-sectional) or within subjects (longitudinal). Practically, this means that we include two predictors representing age into the model: one representing the average age per infant (from here on called within-infants age) and the other being the difference between the date that the observation was made (from here on called between-infants age) and its average age. Because we assumed that over the course of ontogeny, infants would take a more active role we also included the two two-way interactions between role and the two variables representing infant age into the first three models. To control for confounding effects, we also included party composition, infant sex and study site as further fixed effects. As random effects (intercepts), we included the identity of the mother and the infant into the model. To keep type 1 error rates at the nominal level of 5 %, we also included the random slopes components of role, within-infants age and their interaction as well as carry necessity within infant identity (Barr et al. 2013; Schielzeth and Forstmeier 2009). We did not include any other random slopes components within mother ID because with a single exception each mother only had a single infant and hence random slopes of these fixed effects within mother ID would be highly redundant. For the other fixed effects, we did not include random slopes because they were most usually constant within mother and infant ID. We also did not include correlations between random slopes and random intercepts in order to keep model complexity at an acceptable level and because neglected random slopes do not compromise type 1 error rates (Barr et al. 2013). The models were implemented in R (R Core Team 2014) using the function *glmer* of the package ‘lme4’ (Bates et al. 2014). To test the overall significance of our key test predictors (Forstmeier and Schielzeth 2011; Mundry 2014), we compared the full models with the null models comprising only the two control predictors with fixed effects as well as all random effects using a likelihood ratio test (Dobson 2002). Prior to running the models, we *z*-transformed between-infants age, within-infants age and parity (Aiken and West 1991; Schielzeth 2010). To control for collinearity, we determined variance inflation factors (VIF; Field 2005; Quinn and Keough 2002) from a model including only the fixed main effects using the function *vif* of the R package ‘car’. This revealed collinearity to not be an issue (maximum VIF = 1.44). To estimate model stability, we excluded the levels of random effects one at a time, ran the models again and compared the estimates derived with those obtained from the models based on all data. This revealed all models to be at least ‘moderately’ stable, particularly for those estimates that were not close to zero (for details on model stabilities, see supplementary material in Online Resource 2). Confidence intervals were derived using the function *sim* of the R package arm (Gelman and Su 2014). Tests of the individual fixed effects were derived using likelihood ratio tests (R function *drop1* with argument ‘test’ set to ‘Chisq’). All statistical analyses were performed using the R-version R.3.1.1 (R Core Team 2014), with a level of significance set to 0.05.

## Results

**Fig. 1:**
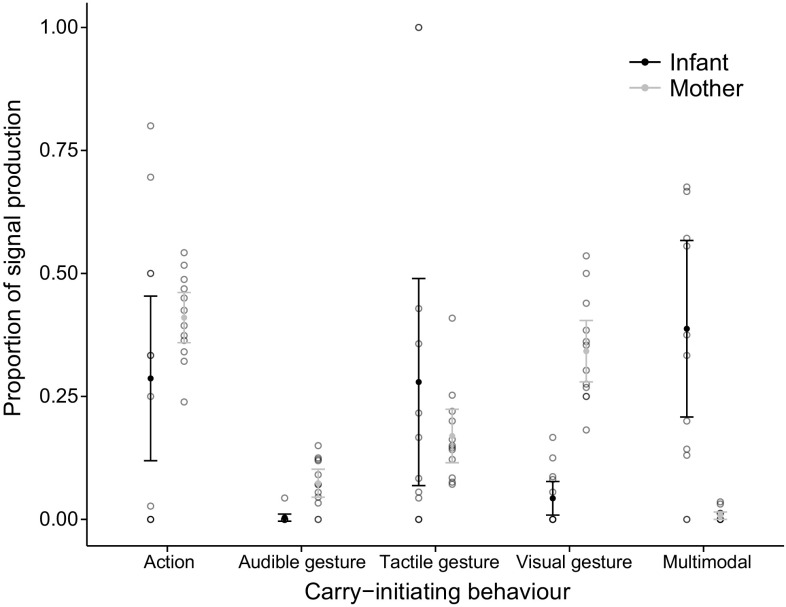
Proportion of carry-initiating actions, gestures (audible, tactile and visual) and multi-modal combinations produced by infants (*N* = 12) and mothers (*N* = 12), respectively. *Error bars* depict the mean values and the 95 % confidence intervals

During 156 days of observation, we recorded a total of 145.2 h of video footage on mother–infant interactions (*Kanywara*: 80.4 h, 11.5 ± 4.7 h per dyad; *Taï South*: 64.8 h, 10.8 ± 5.8 h per dyad; mean ± S.D.). This method resulted in a total of 415 recordings of mother–infant carry initiations (*Kanywara*: *N* = 218; 31.1 ± 13.5 per dyad; *Taï South*: *N* = 197, 32.8 ± 21 per dyad). The coding of this data set resulted in a total (number of cases) of 442 actions (*Kanyawara* mothers: *N* = 178, infants: *N* = 20; *Taï* mothers: *N* = 204, infants: *N* = 40), 599 gestures (*Kanyawara* mothers: *N* = 337, infants: *N* = 22; *Taï* mothers: *N* = 228, infants: *N* = 12), 51 multi-modal combinations (*Kanyawara* mothers: *N* = 2, infants: *N* = 28; *Taï* mothers: *N* = 4, infants: *N* = 17) and 80 vocalisations (*Kanyawara* mothers: *N* = 3, infants: *N* = 39; *Taï* mothers: *N* = 6, infants: *N* = 32). Hence, across both study sites, chimpanzee mothers produced the bulk of gestures and actions, while infants produced gestures less often to initiate joint travel, but most multi-modal combinations and vocalisations (Fig. 1). Results showed that mothers initiated the majority of observed joint travel events at both study sites (*Kanyawara*: 153 out of 218 events; 70.2 %; *Taï:N* = 119 out of 197 events; 60.4 %). In seven cases could the carry initiator not be clearly determined.

### Assessing the influence on sampling size

To ensure that our assessment of individuals’ repertoires had approached and/or reached asymptote, we plotted the cumulative repertoire of gestures over time. The results showed that the cumulative repertoire of mothers approached an asymptote at around the first third of the observation period (see Online Resource 1, Fig. S1). Except for two individuals (MB and JL of *Taï South*, WA of *Kanyawara* community) showed the latest observed gesture type of their repertoire within the first two thirds (67 %) of their total observation time, i.e. within 61.7 ± 28.8 h of full observation (mean ± SD). During the follow-up seasons, only two additional gestures were recorded (*Taï South* in 2013). Concerning the gestural repertoires of these ten chimpanzee mother–infant dyads, there was no correlation between the observed time for each dyad and the final gestural repertoire of each individual (mothers: Spearman’s *R* = 0.494, *P* = 0.147; infants: Spearman’s *R* = 0.253, *P* = 0.48). Thus, we concluded to have observed the full gestural repertoires employed by ten out of 13 dyads (i.e. 20 individuals) in our respective context and study periods. Consequently, for the analyses of the within- and between-group concordance rates, only data from these individuals were used.

### Signal repertoires in carry interactions

To investigate our first question concerning behaviours that chimpanzees employ to initiate joint travel, we analysed actions, gestures, multi-modal combinations and vocalisations for mothers and infants of each site separately. Concerning gestures types, mothers showed a total of one and two audible, each ten visual, six and five tactile gesture types at *Kanyawara* and *Taï South*, respectively (total and mean individual repertoire size *Kanyawara*: 17; mean ± SD = 10 ± 3.7, *N* = 7; *Taï South*: 17; 10.2 ± 4.1, *N* = 5; Table 3a). Idiosyncratic gestures were performed by three different mothers (i.e. one and two adult females from each *Kanyawara* and *Taï South*, each observed more than 112 h) and were termed shakeback, turnbipedal and rearup (see Table 2 for descriptions). Multi-modal combinations in mothers consisted of the vocalisation softhoo with one of the following gestures: presentback (visual, observed in OT of *Kanyawara*) and reacharm (visual, observed in MB and IS of *Taï South,* Table 3b).

**Table 3:** Carry-initiating behaviours, i.e. types of (a) gestures and (b) actions, vocalisations and multi-modal combinations, produced by chimpanzee mothers of both sites (*Kanyawara* [K]: *N* = 7; *Taï South* [T]: *N* = 5) in respective study periods

Age	ID/Study Period	Site	Audible gestures			Tactile gestures							Visual gestures													Total
			ls	so	Total	pl	pu	pv	rt	sb	to	Total	bs	el	lb	ld	lo	lr	pb	ps	pv	ra	ru	tb	Total	–
(*a*)																										
9–11	WA/1	K	X		1	X	X	X	X		X	5			X		X		X		X				4	10
10–12	IS/2	T			0		X	X	X		X	4		X	X		X		X		X		X		6	10
10–12	MB/1	T	X		1			X	X		X	3		X	X		X	X	X			X			6	10
14–16	ML/1	K	X		1	X	X				X	3					X		X						2	6
14–16	OT/1	K	X		1		X	X			X	3		X	X	X	X		X			X			6	10
15	JL	T	X		1		X				X	2			X				X						2	5
15–17	SM/1	T	X		1		X	X				2		X					X	X					3	6
16–18	LN	K	X		1	X		X			X	3			X				X			X			3	7
16–19	TG/1	K	X		1		X	X	X	X	X	5		X	X	X	X	X	X	X	X	X			9	15
19–21	KS/1	T	X		1			X	X		X	3			X		X			X		X			4	8
20–24	WA/2	K	X		1				X		X	2		X	X				X	X		X			5	8
22–24	MB/2	T		X	1	X	X	X			X	4		X	X		X	X	X	X	X	X		X	9	14
26–28	ML/2	K	X		1	X	X	X			X	4					X		X						2	7
26–28	OT/2	K	X		1	X		X			X	3	X	X	X		X		X		X	X			7	11
27–29	SM/2	T	X		1		X	X			X	3		X	X		X		X		X				5	9
28–30	TG2	K	X		1		X		X	X	X	4		X	X		X		X		X				5	10
31–33	KS/2	T	X		1		X	X	X		X	4			X		X				X	X			4	9
48–50	OU	K	X		1				X			1		X	X				X						3	5
55–56	WL/1	K	X		1				X			1	X	X	X		X		X	X	X				7	9
64–65	IS/1	T			0	X						1			X		X					X			3	4
67–69	WL/2	K	X		1				X		X	2	X	X	X		X		X			X			6	9

Age	ID/Study Period	Site	Action						Vocal	Multi-modal
			apr	frz	grb	mov	lob	Total	sgr	sgr + gesture
(*b*)										
9–11	WA/1	K	X		X	X		3		
10–12	IS/2	T	X	X	X	X	X	5	X	ra
10–12	MB/1	T			X	X		2	X	ra
14–16	ML/1	K			X			1		
14–16	OT/1	K	X		X	X		3		
15	JL	T		X	X	X	X	4		
15–17	SM/1	T				X	X	2		lo
16–18	LN	K	X	X		X		3	X	
16–19	TG/1	K	X		X	X		3		
19–21	KS/1	T	X	X		X	X	4	X	
20–24	WA/2	K	X	X	X	X		4		
22–24	MB/2	T	X	X		X	X	4		
26–28	ML/2	K		X		X		2		
26–28	OT/2	K	X	X		X	X	4		pb
27–29	SM/2	T	X	X		X		3		
28–30	TG/2	K		X		X		2		
31–33	KS/2	T	X	X		X	X	4		
48–50	OU	K		X		X		2		
55–56	WL/1	K	X	X		X		3		
64–65	IS/1	T	X			X		2		
67–69	WL/2	K	X	X		X		3		

Compared with chimpanzee mothers, infants had significantly smaller gestural repertoires (*t* = 7.993, *df* = 18, *P* < 0.001; Levene’s test for variance equality: *Z* = 2.424, *P* = 0.137), producing one and zero audible, four and two tactile; and three visual gesture types at *Kanyawara* and *Taï South,* respectively (total and mean individual repertoire size at *Kanyawara*: 8; mean ± SD = 2.3 ± 1.8, *N* = 7; *Taï*: 5; 2.5 ± 1.6, *N* = 6; Table 4a). All gesture types except for one tactile gesture (armon) that was produced by two older infants (WC and OL) at *Kanyawara* were shared with the mothers (Table 2). Multi-modal combinations in infants consisted of the vocalisation hoowhimper with one of the following gestures: touch (tactile, observed in three infants: MH, TR and WC), lookat (visual, observed in nine infants: IN, IT, KY, MH, OL, SL, TR, WZ), reacharm (visual, observed in four infants: IN, KY, MH, TR) or loudscratch (audio-visual, observed in one infant: WC; Table 4b). While there were more visual gesture types and combined forms of gestures and vocalisations observed in older infants (i.e. infants from the second year of life, Table 4b), final gestural repertoire size in both mothers and infants was not significantly correlated with final infant age (mothers: Spearman’s *R* = −0.037, *P* = 0.920, *N* = 10; infants: Spearman’s *R* = 0.544, *P* = 0.104, *N* = 10). Naturally, due to the obvious asymmetry in the carry interaction (Halina et al. 2013) repertoires of chimpanzee infants were more similar to each other than repertoires of mothers. Since the sample sizes of audible gestures and multi-modal signals were comparably low, no inferential statistics has been conducted on this gestural category.

**Table 4:** Carry-initiating behaviours, i.e. types of (a) gestures and (b) actions, vocalisations and multi-modal combinations, produced by chimpanzee infants of both sites (*Kanyawara* [K]: *N* = 7; *Taï South* [T]: *N* = 6) in respective study periods

Age	ID/study period	Site	Audible gestures		Tactile gestures					Visual gestures				Total
			sc	Total	ao	to	pl	pu	Total	lo	lb	ra	Total	–
(*a*)														
9–11	WZ/1	K		0			X		0	X		X	2	2
10–12	IN	T		0		X			1	X		X	2	3
10–12	MH/1	T		0		X			1			X	1	2
14–15	MM/1	K		0		X			1				0	1
14–16	OB/1	K		0					0				0	0
15	JF	T		0					0				0	0
15–17	SL/1	T		0		X			1				0	1
16–18	LL	K		0		X			1				0	1
16–19	TR/1	K	X	1					0		X	X	2	3
19–21	KY/1	T		0		X			1				0	1
20–24	WZ/2	K		0					0				0	0
22–24	MH/2	T		0		X			1	X	X	X	3	4
26–28	MM/2	K		0					0				0	0
26–28	OB/2	K		0					0				0	0
27–29	SL/2	T		0	X	X			2	X			1	3
28–30	TR/2	K		0		X			1			X	1	2
31–33	KY/2	T		0					0	X	X	X	3	3
48–50	OL	K		0	X			X	2	X			1	3
55–56	WC/1	K		0	X	X			2	X			1	3
64–65	IT	T		0					0	X			1	1
67–69	WC/2	K	X	1	X	X	X		3	X			1	5

Age	ID/Study period	Site	Action					Vocal	Multi-modal
			apr	frz	grb	hon	Total	whi	whi + gesture
(*b*)									
9–11	WZ/1	K	X			X	2	X	lo
10–12	IN	T	X			X	2	X	lo, ra
10–12	MH/1	T	X			X	2		
14–15	MM/1	K					0	X	
14–16	OB/1	K	X			X	2		
15	JF	T	X				1	X	
15–17	SL/1	T	X				1		
16–18	LL	K	X				1	X	
16–19	TR/1	K	X	X		X	3	X	lo
19–21	KY/1	T	X	X		X	3	X	
20–24	WZ/2	K	X			X	2	X	
22–24	MH/2	T	X			X	2	X	lo, ra, to
26–28	MM/2	K	X				1	X	
26–28	OB/2	K	X	X		X	3	X	
27–29	SL/2	T	X	X	X	X	4	X	lo
28–30	TR/2	K	X			X	2	X	to, ra
31–33	KY/2	T	X	X	X	X	4	X	lo, ra
48–50	OL	K					0	X	lo
55–56	WC/1	K	X			X	2	X	lo
64–65	IT	T	X				1	X	lo
67–69	WC/2	K					0	X	lo, sc, to

### Within- and between-group concordance of mother’s carry-initiating gestures

To address the second question on whether gesture types produced to initiate joint travel are learned during mother–infant exchanges or due to genetic channelling, we calculated the rate of concordances (repertoire similarity) within and between groups by using the Dice coefficient (D_*c*_). Overall, D_*c*_ values were moderate, irrespectively which community the individuals belonged to (overall: D_*c*_ = 0.71 ± 0.1, mean ± SD; *Kanyawara*: D_*c*_ = 0.71 ± 0.1; *Taï South:* D_*c*_ = 0.71 ± 0.05; see Online Resource 1, Table S3). Comparing the concordance rates of mothers of the same and the other community, we did not find a significant difference between the within-group (D_*c*_ = 0.71 ± 0.1) and the between-group Dice coefficients (D_*c*_ = 0.71 ± 0.1; matrix permutation: *P* = 0.839).

### Factors influencing signal production and carry initiations

To examine the third question on whether infant age influenced the behaviours used to initiate joint travel, we ran four different models. Overall, the test predictors had a clear impact in all models, i.e. on the occurrence of actions, tactile and visual gestures as well as on the role of joint travel initiator [likelihood ratio tests (LRT) comparing null and the full model for action: *χ*^*2*^ = 23.476, *df* = 7, *P* = 0.001, tactile gesture: *χ*^*2*^ = 18.968, *df* = 7, *P* = 0.008, visual gesture: *χ*^*2*^ = 52.795, *df* = 7, *P* < 0.001, carry initiator: *χ*^*2*^ = 24.320, *df* = 4, *P* < 0.001].

**Fig. 2:**
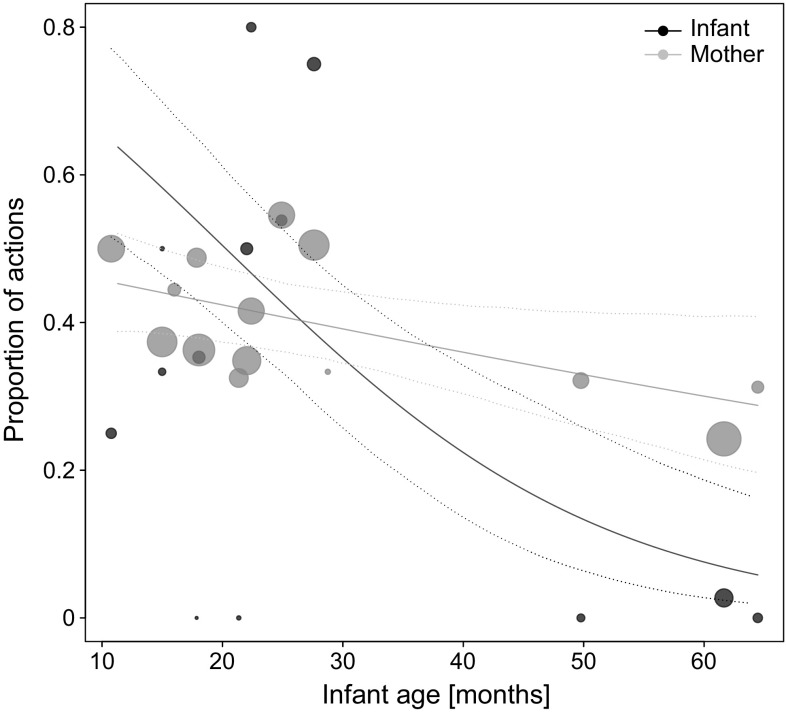
Proportion of actions employed to initiate joint travel as a function of dyadic role and infant age. Depicted are proportions, separately for each mother and infant of a dyad against mean infant age. The area of the *dots* corresponds to the sample size per individual (range 1–132); the *solid* and *dashed lines* represent the fitted model and confidence intervals based on all covariates and factors centred to a mean of zero

Concerning *carry*-*initiating actions*, we found a significant interaction between role and between-infants age (estimate ± standard error = 0.756 ± 0.250, $\chi^{2}_{1}$ = 11.668, *P* = 0.002), with younger infants and mothers of younger infants soliciting more frequently joint travel via actions than older infants (Fig. 2). None of the other effects reached significance (Table 5a).

**Table 5:** Factors influencing (a) action production, (b) tactile gesture production, (c) visual gesture production and (d) initiator of carry initiation in mother–infant dyad

	Estimate	se	*χ* ^2^	*P*	LRT *χ* ^2^	*df*	*P*
*(a) Action*							
Intercept	−0.758	0.318	^(a)^	^(a)^	23.476	7	**0.001**
Role (mother)	0.217	0.207	^(a)^	^(a)^			
Within-infants age	−0.185	0.201	^(a)^	^(a)^			
Between-infants age	−0.988	0.248	^(a)^	^(a)^			
Carry necessity	0.009	0.154	0.003	0.953			
Parity	0.007	0.096	0.005	0.942			
Party (females)	−0.042	0.24	0.031	0.861			
Party (mixed)	−0.127	0.237	0.287	0.592			
Infant sex (male)	0.019	0.185	0.010	0.919			
Site (Taï)	0.407	0.195	3.705	0.054			
Role: within-infants age	0.215	0.223	0.924	0.336			
**Role: between-infants age**	**0.756**	**0.25**	**11.668**	**0.001**			
*(b) Tactile gesture*							
Intercept	−1.437	0.383	^(a)^	^(a)^	18.968	7	**0.008**
Role (mother)	0.065	0.258	0.064	0.800			
Within-infants age	−0.164	0.089	2.824	0.093			
Between-infants age	0.049	0.136	0.139	0.709			
**Carry necessity**	−**0.649**	**0.210**	**9.861**	**0.002**			
Parity	−0.019	0.11	0.030	0.863			
Party (females)	0.274	0.331	0.683	0.409			
Party (mixed)	0.149	0.299	0.253	0.615			
Infant sex (male)	0.006	0.218	0.001	0.979			
**Site (Taï)**	−**0.646**	**0.233**	**5.479**	**0.019**			
*(c) Visual gesture*							
Intercept	−3.134	0.428	^(a)^	^(a)^	52.795	7	**<0.001**
**Role (mother)**	**2.380**	**0.359**	**46.963**	**<0.001**			
Within-infants age	−0.006	0.106	0.003	0.957			
Between-infants age	0.182	0.090	2.973	0.085			
**Carry necessity**	**0.436**	**0.163**	**5.785**	**0.016**			
Parity	0.109	0.082	1.760	0.185			
Party (females)	−0.148	0.252	0.343	0.558			
Party (mixed)	0.122	0.245	0.250	0.617			
Infant sex (male)	−0.069	0.16	0.187	0.665			
Site (Taï)	0.029	0.168	0.029	0.864			
*(d) Infant carry initiation*							
Intercept	−1.944	0.476	^(a)^	^(a)^	24.320	4	**<0.001**
Within-infants age	0.547	0.242	3.297	0.069			
**Between-infants age**	**0.779**	**0.163**	**16.235**	**<0.001**			
Carry necessity	0.004	0.286	0.000	0.990			
**Parity**	**0.651**	**0.148**	**11.601**	**0.001**			
Party (females)	0.912	0.482	3.836	0.050			
Party (mixed)	0.768	0.470	2.807	0.094			
Infant sex (male)	0.027	0.297	0.008	0.928			
Site (Taï)	0.468	0.286	1.923	0.165			

Bold values indicate *P* < 0.05

^(a)^Significance test not indicated because it has no meaningful interpretation

For *tactile gesturing*, we found none of the two interactions (role interacting with both between-infants and within-infants age) to be significant. After removal of these non-significant interactions, we found that chimpanzee mothers were less likely to produce tactile gestures with increasing infant age (within-infants age: −0.164 ± 0.089, $\chi^{2}_{1}$ = 2.824, *P* = 0.093) and produced them more frequently for less urgent carries (carry necessity: −0.649 ± 0.210, $\chi^{2}_{1}$ = 9.861, *P* = 0.002). In addition, individuals of the *Kanyawara* community produced tactile gestures more often than chimpanzees from the *TaïSouth* community (site [Taï South]: −0.646 ± 0.233, $\chi^{2}_{1}$ = 5.479, *P* = 0.019). None of the other effects reached significance (Table 5b).

**Fig. 3:**
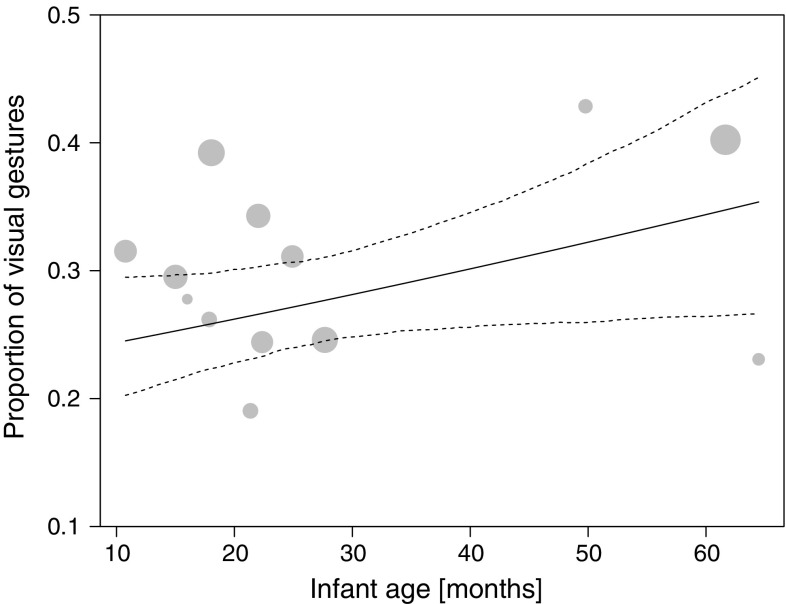
Proportion of visual gestures employed to initiate joint travel as a function of infant age. Depicted are proportions, separately for each dyad against the respective mean infant age. The area of the *dots* corresponds to the signal sample size per mother–infant dyad (range 20–171); the *solid* and *dashed line(s)* represent the fitted model and confidence intervals based on all covariates and factors centred to a mean of zero

**Fig. 4:**
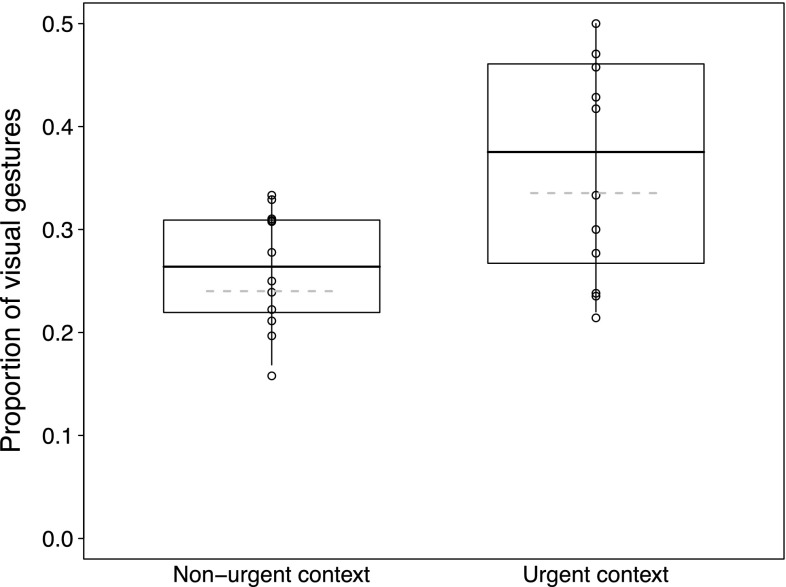
Proportion of visual gestures employed to initiate joint travel as a function of carry necessity. The *dashed lines* represent the fitted model (conditional on all covariates and factors centred to a mean of zero)

In the *visual gesturing* model, we also found none of the two interactions to be significant. After removal of these non-significant interactions, the results showed that chimpanzee mothers were more likely to produce visual gestures than infants (role [mother]: 2.380 ± 0.359, $\chi^{2}_{1}$ = 46.963, *P* < 0.001). In dyads involving older infants, mothers had a higher frequency of producing visual gestures than dyads involving younger infants (between-infants age: 0.182 ± 0.090, $\chi^{2}_{1}$ = 2.973, *P* = 0.085, Fig. 3). In addition, visual gestures were produced more frequently when carries were more necessary (carry necessity: 0.436 ± 0.163, $\chi^{2}_{1}$ = 5.785, *P* = 0.016, Fig. 4). None of the other effects in the visual gesture model reached significance (Table 5c).

In the fourth model, we examined which factors influenced whether mothers or infants *initiated carries for joint travel*. After removal of the non-significant interactions, we found a strong effect of infant age: with increasing age, infants initiated more carries (within-infants age: 0.547 ± 0.242, $\chi^{2}_{1}$ = 3.297, *P* = 0.069; between-infants age: 0.779 ± 0.163, $\chi^{2}_{1}$ = 16.235, *P* < 0.001). In addition, in dyads with mothers of higher parity, mothers were less likely to initiate carries (0.651 ± 0.148, $\chi^{2}_{1}$ = 11.601, *P* < 0.001). None of the other effects reached significance (Table 5d).

## Discussion

The main aim of the present study was to gain a better understanding of the complexity and variability of communicative exchanges in chimpanzee mother–infant dyads in natural environments and to shed light on gestural acquisition. Since previous studies on gestural variability have emphasised the importance of long-term observations to reliably assess repertoire size, we observed the communicative behaviour of mother–infant dyads of two chimpanzee communities during two field periods in two consecutive years for more than 150 days, and examined the cumulative frequency of gesture type. The results showed that the rate of adding new gestures to the repertoires of our focal animals of the *Kanyawara* and *Taï South* community in the single context of joint travel appeared close to asymptote. Further observations are thus unlikely to contribute many additional gesture types.

We addressed the following three questions: First, which behaviours do chimpanzees employ to initiate joint travel? Second, are gesture types produced to initiate joint travel due to learning or are they the result of genetic channelling? Third, do chimpanzee mothers adjust their behaviour to the developmental stage of their infants, and how does infant age influence signal production in both mothers and infants?

Overall, we found striking differences between mothers and infants concerning the signal frequency and modality employed to initiate joint travel. While mothers were the main initiators of joint travel and mainly relied on gestures to do so, infants solicited joint travel frequently via actions and vocalisations. Gestural repertoires differed considerably between mothers living in the same community but also between mothers living in different communities. We observed three cases of idiosyncratic gesture performance employed by three different mothers, with one case performed across both study periods. No evidence of community-specific gesture performance was found. Furthermore, the results indicated that infant age and necessity of the carry had a crucial impact on signal production. In the following paragraphs, we will discuss each of our research question and the related findings in detail.

Chimpanzee mothers and infants differed considerably in the behavioural modalities used to initiate joint travel, but also in their communicative tool set, i.e. the variety of actions, gestures and multi-modal combinations employed. This result is in line with our expectations since the asymmetry of the carry interaction predicts ‘one-way’ production of distinct actions and gesture types such as for instance lift on back, backwardsweep and armon (Halina et al. 2013). However, chimpanzee mothers also initiated the majority of all carry instances and showed a much higher frequency and variety of gesture types produced, with maternal repertoires being generally much larger than infant repertoires. These results are in some contrast to a similar study on bonobo gesturing in captivity, with infants actively soliciting joint travel more often and producing a higher variety of gestures (Halina et al. 2013). There are two different explanations: First, differences between the two studies might represent differences in communication styles or the sensitivity to ‘cultural variation’ of bonobos and chimpanzees (Pollick and De Waal 2007). Since the two Pan species seem to differ extensively concerning the risks of infanticide, male harassment and resulting coalition styles (e.g. Boesch 1991; Mitani et al. 2000; Surbeck et al. 2011; van Schaik 1996), strong mother–infant associations and relationships in chimpanzees may have been selected for. This in turn may then have triggered a higher degree of protectiveness and modification of the communicative tool kit. However, we can neither verify nor refute this explanation since to date no quantitative comparisons of mother–infant communication in bonobos and chimpanzees are available. A second and more parsimonious explanation therefore is that differences between the two studies might simply represent different sampling methods applied and diverging ecological environments (i.e. captive versus natural environments). Both bonobos and chimpanzees have to cope in the wild with relatively long travel distances between feeding patches (Furuichi et al. 2008; Pontzer and Wrangham 2004), encountering other group members on a regular basis. Consequently, the maternal style of protectiveness described in captivity—associated with contact-making, approaching and restraining the infant (De Lathouwers and Van Elsacker 2004)—may play an important role over an even more extended time period in natural environments. Intriguingly, our results showed that mothers with higher parity were less likely to initiate joint travel. It seems possible that multiparous mothers, i.e. those that live in the community for several years, act less cautious because they have more experience in evaluating and assessing possible dangers and risks due to previously reared offspring.

An additional important difference between mother and infant signalling concerned the use of the communicative modality: Mothers mainly produced visual gestures, while infants preferred to initiate joint travel via vocalisations (i.e. hoowhimper*)* or multi-modal combinations (i.e. hoowhimper and look; hoowhimper and reach*)*. Especially in older infants from the age of 2 years, vocalisations were frequently used in intentional ways in combination with (mainly visual) gestures. Thus, similarly to some alarm calls of chimpanzees (Schel et al. 2013), whimpering might develop into an intentional signal with the goal of inducing the mother’s interaction through understanding of the signal meaning through its social effect (Plooij 1978). Our study thus adds a new facet to developmental processes in vocal and gestural signalling (Pika et al. 2003; Seyfarth and Cheney 1997) by providing the first evidence that at least in some contexts, a developmental shift from merely vocal to mainly gestural signalling takes place. Furthermore, whimpering in chimpanzee infants seems to gain its communicative intentional function in concert with gestures that function to re-establish physical contact with the mother. Similarly, Goodall (1967) described several clear-cut signals produced by chimpanzee infants that served to re-establish physical contact with the mother (e.g. reacharm, accompanied by pout face and hoowhimper). Hence, when studying the development of communicative skills in chimpanzees and probably also other great ape species, it seems absolutely mandatory to make use of a multimodal approach to communicative complexity (e.g. Slocombe et al. 2011).

Although it has been shown numerous times that great apes use gestures in intentional and flexible ways and are able to acquire novel gesture types (Pika 2015), it remains controversial how great ape repertoires are acquired. In the most predominant hypothesis, gestures are learned via OR (Tomasello et al. 1994), while another hypothesis postulates that gestural production is innate, leaving no room for modification of form over time but including flexible use across contexts (Genty et al. 2009; Hobaiter and Byrne 2011). To contribute to this debate, we carried out the first systematic comparison of communicative exchanges in mother–infant dyads living in two different chimpanzee communities. We paid particular attention to the main criticisms raised by Byrne and colleagues (Genty et al. 2009; Hobaiter and Byrne 2011) on captive studies (e.g. the definition of idiosyncracy and shortage of observational periods). Our results showed only moderate levels of concordance rates between the individual gestural repertoires of mothers living in the same community but also between subspecies and communities. We did not find any evidence for subspecies/community-specific gesture production, but observed three distinct gesture types, which were produced by single mothers only (rearup, turnbipedal and shakeback) across both study periods. The gestural repertoires of these three females had approached an asymptote within the first observation period. A detailed review of the ethograms of two long-term studies of chimpanzee behaviour (Goodall 1986; Nishida et al. 1999) and several gesture studies (Call and Tomasello 2007; Hobaiter and Byrne 2011; Roberts et al. 2014a) did not produce any comparable behaviours in any other chimpanzee community or group. There are three possible explanations: First, gestural production can be explained by genetic channelling only (Genty et al. 2009; Hobaiter and Byrne 2011). If this hypothesis were true, then we would have expected to find high levels of gestural concordances within and between groups and no evidence for idiosyncracy. This prediction does not accord with our observations. Second, gestural production is due to genetic channelling with gestural variability between groups representing genetic dissimilarity of two subspecies. If this hypothesis were true, then we would have expected to find high degrees of gestural concordances within groups but not between groups, which also does not accord with our observation. However, since evidence of high degrees of gestural concordances within single communities does not enable to differentiate between the processes of genetic channelling or social learning (Bandura 1986), investigations of gestural production of several communities of eastern and/or western African chimpanzees would have been compulsory. Nevertheless, although systematic investigations of gestural signals have so far mainly been focusing on wild communities of Eastern African chimpanzees (Goodall 1986; Hobaiter and Byrne 2011; Nishida et al. 1999; Roberts et al. 2014a), the majority of studies on chimpanzee behaviour provide evidence for considerable inter-site variability rather than differences between sub-species including communicative signalling (e.g. Boesch et al. 1994; Whiten et al. 1999). Third, gestures produced during mother–infant interactions are due to learning. Consistent with this hypothesis is the finding of gestural variability, with moderate levels of concordances in the class of mothers within and between groups. Furthermore, we found clear evidence for the production of three idiosyncratic gesture types produced by three different females, which, to our knowledge, have not been described by other researchers. Since we applied even more conservative criteria than previous studies producing similar findings concerning the acquisition of gestures in great apes (Call and Tomasello 2007; Halina et al. 2013; Roberts et al. 2014a), we conclude that indeed learning plays a crucial role in gestural acquisition. However, to address the question which gesture types are more prone to be acquired (e.g. Bard et al. 2014) and which exact details are picked up upon, new methodological tools and fine-grained analyses are crucial (Perlman et al. 2012). Furthermore, we postulate a revised theory of gestural acquisition, ‘social negotiation’, because the theory of OR (a) is in our view not a completely satisfactory explanation and (b) has led to several misconceptions. First, since it postulates that a physically effective *sequence of actions* is ‘ritualised’ into a communicative signal (Tomasello et al. 1994), several researchers have tried rather unsuccessfully to identify these action sequences (Genty et al. 2009; Hobaiter and Byrne 2011). Second, it is widely assumed that gestures acquired via OR cannot be generalised across dyads, resulting in one-way gestures, idiosyncratic repertoires (Genty et al. 2009; Halina et al. 2013; Tanner et al. 2006) and thus no shared meaning within communities. This is beside the evidence that chimpanzees and bonobos (a) are able to use referential gestures and ideograms across contexts and experimenter (e.g. Gardner and Gardner 1991; Savage-Rumbaugh et al. 1986), (b) utilise some group-specific gestures which carry different meanings across groups (for an overview see Pika et al. 2005) and (c) understand the goals and intention of others as well as third-party relationships (Call and Tomasello 2008; Mitani et al. 2000). They thus clearly possess the cognitive abilities to also generalise established communicative value and meaning of gestures across dyads in their natural communication with conspecifics (for evidence concerning continuity of gestural repertoire across time and interaction partners in gorillas, see Tanner 1993).

The redefined theory of *social negotiation* (sensu Plooij 1978; Wittgenstein 1953) thus proposes that the creation of gestures does not begin with shaping and shortening of a functional action sequence, but an exchange of social behaviours resulting in a shared understanding that certain behaviours (a) can be used communicatively, (b) carry distinct meaning linked to particular social contexts and (c) are produced to achieve distinct goals. This knowledge can be generalised across dyads to enable the most efficient and least costly communication transfer but is also open to subsequent adaptation (e.g. a gesture might first be used to initiate play but later to impress a possible rival). In line with this theory, Bard et al. (2014) recently proposed that most gestures emerge from meaningful social interactions through inter-subjective processes, vary according to the context (Fogel and Thelen 1987) and may rely on ‘continued communicative validation’. While the form of gestures is indeed naturally constrained by anatomical features and movement restrictions of a given species (sensu Hobaiter and Byrne 2011), but also the communicative scenario (e.g. short-distance communication versus long-term communication, interaction partner), social context (Wittgenstein 1953) and recipient-affordances (attentional state, location, posture and distance to recipient; Pika 2014), the outcome is ‘mutually shaped’ (King 2004) or in our words ‘social negotiated’ by interactants in real time. The resulting gestural output is a manifold variation concerning manner, size, scope, strength, location and orientation of gesture. For instance, although researchers concordantly embrace light and brief (under 2 s) contact of the palm and/or fingers of signallers on the body of the recipient under the single umbrella term touch, each gestural performance of a touch gesture by a given signaller is a highly variable online adjustment (Perlman et al. 2012). Additional, in-depth studies of ape gestural production are needed to investigate the form of gestures in relation to developmental phase, context and interaction partner.

Concerning developmental trends and the question whether mothers adjusted their gestural communication to the developmental stage of their infants, we found that visual gestures were employed more frequently with increasing infant age, while the production of tactile gestures decreased. In addition, carry-initiating actions were produced more frequently by dyads with younger infants and decreased considerably with progressing development. Moreover, older infants initiated more carries than younger infants. These findings are in line with our expectations since with increasing age, chimpanzee infants quite naturally increase the distance to their mothers and become intentional agents, who manipulate the attentional and maybe also the mental states of their conspecifics (Pika and Mitani 2006; Plooij 1979; Tomasello et al. 2003). Our findings thus support the notion of Goodall (1967), who suggested that chimpanzees’ communicative development may rely heavily on the infant leaving the ‘security range’ of the mother and entering the complex social environment. As physical distance between mothers and their maturing infants increases, visual gestural communication, in addition to vocalisations, becomes the most crucial communicative modality for mother–infant coordination (Bard et al. 2005; van Lawick-Goodall 1967). With regard to behaviours that were used to initiate joint travel in mother–infant dyads of different study sites, we found no group-specific patterns of carry initiations. Observed patterns at both sites were consistent with anecdotal observations concerning gesture types (e.g. extendleg, lookback, presentback) and use of multimodal signals (e.g. hoowhimper + reacharm) reported from Gombe (Goodall 1986; Plooij 1978; van Lawick-Goodall 1967). However, since we systematically addressed the communicative function of carry initiations, our study revealed many more gestures types and thus enabled a more detailed understanding of the variability of carry-initiating actions, gestures and vocalisations employed for this single communicative function.

Surprisingly, our results revealed that visual, but not tactile, gestures were frequently produced in ‘evolutionarily urgent’ situations, i.e. contexts that underlie strong selection pressure, such as catching-up with an already left party, aggression and group travel. Our findings thus question the hypothesis of Tomasello and Zuberbühler (2002), proposing that primate gestural communication shows more flexibility than primate vocal communication due to gestures being employed in less evolutionary urgent contexts. Quantitative comparisons of the frequency of vocal and gestural production with respect to context urgency have, however, not been carried out. The only exemption is the study by Hobaiter and Byrne (2012), which showed that male chimpanzees preferred to use gestures rather than vocalisations in the evolutionary urgent context of consortship. Gestural communication might therefore outcompete vocal signalling in those contexts when the risk of alerting other group members (e.g. consortship), members of other communities (e.g. patrol) or possible predators is relatively high (however, see Crockford et al. 2012 for usage of soft calls). The employment of visual gestures by mothers might be an adaptive strategy to signal efficiently when a carry would be rather urgent, e.g. when potentially dangerous males arrive or when the party has already left. Contrarily, in non-urgent preceding situations such as feeding via travelling from tree to tree, it might be less crucial for a mother to actively gesture her intention to leave, since her body is indicating the main travel direction and the infant can decide whether to simply follow by himself or whether to climb aboard (Nishida et al. 1999).

In sum, the present study has shown that chimpanzees employ a variety of different behaviours to initiate mother–infant joint travel, with a developmental shift from mainly vocal to gestural signalling and adjustment of mothers to the developmental stage of infants. Applying a windows approach onto communicatory signalling can therefore crucially aid in gaining an in-depth understanding of the communicative tool kit of a given species. Furthermore, by making the first step into the crucial direction of systematic quantitative comparisons of communicative signalling between different chimpanzee subspecies and communities in natural environments, we showed that gestures to initiate joint travel do not represent simple innate, fully formed means, but are the result of underlying learning processes. We thus hope to inspire future studies, testing the social negotiation hypothesis and investigating whether gestural acquisition indeed involves shared understanding and mutual construction in real time by both interactants.

## Electronic supplementary material

Below is the link to the electronic supplementary material.

Supplementary material 1 (PDF 130 kb)

Supplementary material 2 (PDF 144 kb)

## Footnotes

1 From now on, gesture and vocalisation types are depicted in small capitals.
