# Supplementary material for: Should I stay or should I go? Initiation of joint travel in mother–infant dyads of two chimpanzee communities in the wild
Source: Anim Cogn. 2016 Feb 1;19:483–500. doi: 10.1007/s10071-015-0948-z (PMC4824811; doi:10.1007/s10071-015-0948-z)
Supplement: Supplementary file 1 — Supplementary material 1 (PDF 130 kb) [file 10071_2015_948_MOESM1_ESM.pdf]

Online Resource 1: Additional Tables and Figures

Journal: Animal Cognition

Title: 'Should I stay or should I go?' – Initiation of joint travel in mother-infant dyads of two chimpanzee communities in the wild

Authors: Marlen Fröhlich*, Roman M. Wittig, Simone Pika

Affiliation of corresponding author*: Max Planck Institute for Ornithology, Humboldt Research Group 'Comparative Gestural Signalling',

Eberhard-Gwinner-Strasse 9, 82319 Seewiesen, Germany

Affiliation of corresponding author*: mfroehlich@orn.mpg.de

Table S1 Action types produced to initiate carries in chimpanzee mother-infant dyads identified in this study and corresponding behavioural patterns of the Mahale chimpanzees [46]

Action	Definition (this study)	Used by	Mahale
**Tactile**			
Grab [grb]	Individual grasps or tugs on limbs of another	Both	Grab
Hold on [hon]	Individual clings with one or both hands to fur of another	Infant	Grasp and push shoulders
Lift on back [lob]	Individual lifts infant on own back	Mother	Put dorsal
**Visual**			
Approach [apr]	Individual moves towards another	Both	Approach
Freeze [apr]	Individual pauses movement for > 2 seconds	Both	Wait for companion
Move away	Individual moves away from another	Both	Depart

Table S2 Infant scan data recorded in 15-minute intervals to complement video recordings

Recorded parameter	Levels
1. General	infant ID, date & time, visibility
2. Body posture	cling ventral, cling dorsal, cling side, ride, sit, lie (on conspecific), climb, hang, walk, stand bipedal/quadrupedal, run
3. Activity	breast-feed, feed, rest, play, groom, being groomed, self-groom, exploration, being carried, follow mother, explore, observe others
4. Play type (if applicable)	solitary play, object play (solitary), object play (social), rough play, soft play
5. Object involved type (if applicable)	leaf, stone, stick, bark, fruit(s), plant material, termite mould, liane, water, wood, other
6. Context	feed, rest, play, groom, travel, aggression, patrol, inter-group encounter
7. Interaction partner(s)	ID and relationship of/to conspecific(s)
8. Physical distance between mother and infant	body contact, within arm's reach, 2 arm lengths, 1-2 m, 2-5 m, 5-10 m and >10 m.
9. Attentional state of mother	in visual field, 90°, 180°
10. Group composition	mixed group, only adult females, family (mother and dependent offspring)
11. Height & Vegetation	ground, low-storey, mid-storey, canopy<br>open, moderate, dense

Table S3 Dice coefficients (D_c) measuring the similarity between the gestural repertoires of individual mothers of the same or different chimpanzee group. Initials in *italics* and plain font represent the IDs of mothers from the Taï and Kanyawara groups, respectively.

	IS	***KS***	***MB***	***SM***	**LN**	**ML**	**OT**	**TG**	**WA**	**WL**
IS	1.00									
KS	0.73	1.00								
MB	0.79	0.69	1.00							
SM	0.70	0.67	0.67	1.00						
**LN**	0.56	0.63	0.55	0.43	1.00					
**ML**	0.70	0.67	0.67	0.63	0.71	1.00				
**OT**	0.80	0.70	0.76	0.67	0.63	0.76	1.00			
**TG**	0.74	0.80	0.77	0.70	0.57	0.61	0.79	1.00		
**WA**	0.83	0.82	0.86	0.80	0.67	0.70	0.80	0.89	1.00	
**WL**	0.70	0.76	0.81	0.63	0.75	0.53	0.67	0.77	0.87	1.00

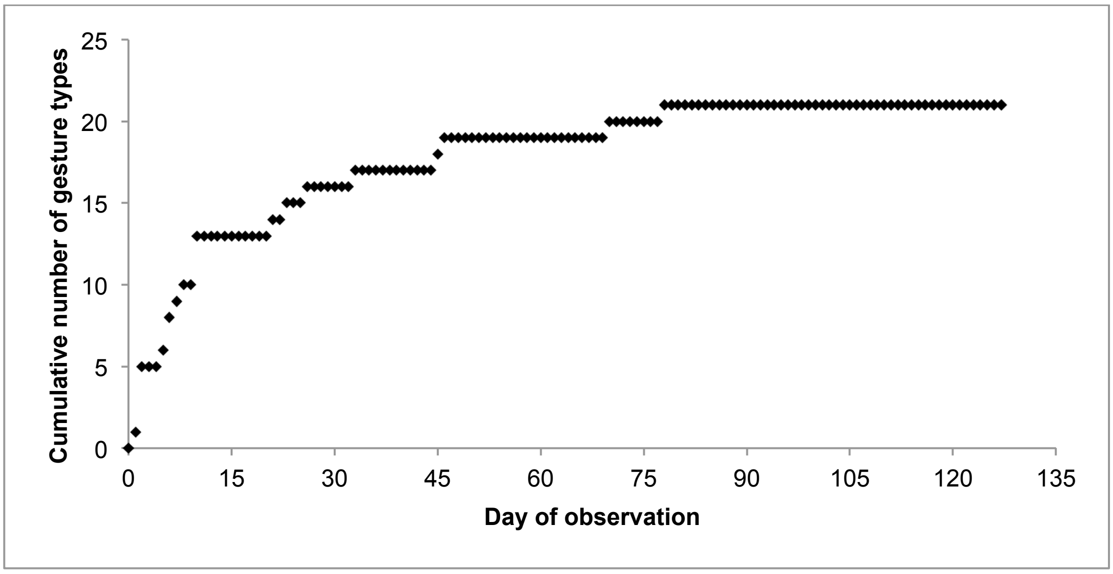

Figure S1 Cumulative gestural repertoire used in joint travel interactions plotted against observation time (days).
