# Supplementary material for: Should I stay or should I go? Initiation of joint travel in mother–infant dyads of two chimpanzee communities in the wild
Source: Anim Cogn. 2016 Feb 1;19:483–500. doi: 10.1007/s10071-015-0948-z (PMC4824811; doi:10.1007/s10071-015-0948-z)
Supplement: Supplementary file 2 — Supplementary material 2 (PDF 144 kb) [file 10071_2015_948_MOESM2_ESM.pdf]

Online Resource 2: Model stabilities

Journal: Animal Cognition

Title: 'Should I stay or should I go?' – Initiation of joint travel in mother-infant dyads of two chimpanzee communities in the wild

Authors: Marlen Fröhlich*, Roman M. Wittig, Simone Pika

Affiliation of corresponding author*: Max Planck Institute for Ornithology, Humboldt Research Group 'Comparative Gestural Signalling', Eberhard-Gwinner-Strasse 9, 82319 Seewiesen, Germany

Affiliation of corresponding author*: mfroehlich@orn.mpg.de

**Action**	orig	min	max
(Intercept)	-0.799	-1.129	-0.624
rolemo	0.202	-0.001	0.459
z.inf_age	-0.171	-0.275	-0.004
z.mean.inf.age	-0.961	-1.303	-0.698
urgency	0.014	-0.034	0.047
inf_sexM	0.032	-0.131	0.195
group.L	-0.089	-0.16	-0.005
group.Q	-0.009	-0.04	0.041
z.mot_exp	0.015	-0.108	0.077
siteT	0.389	0.163	0.658
rolemo:z.inf_age	0.192	-0.022	0.363
rolemo:z.mean.inf.age	0.737	0.528	1.063
infant@role.code	0	0	0
infant.1@z.inf_age	0	0	0
infant.2@urgency	0	0	0
infant.3@int.r.ia	0.203	0.117	0.23
infant.4@(Intercept)	0	0	0.04
mother@(Intercept)	0.213	0.04	0.232

**Tactile gesture**	orig	min	max
(Intercept)	-1.455	-1.808	-0.897
rolemo	0.298	0.023	0.664
z.inf_age	-0.546	-0.685	-0.423
z.mean.inf.age	0.329	0.201	0.438
urgency	-0.797	-0.875	-0.735
inf_sexM	-0.038	-0.243	0.123
group.L	0.136	0.006	0.24
group.Q	-0.221	-0.286	-0.082
z.mot_exp	-0.023	-0.135	0.132
siteT	-0.644	-0.901	-0.418
rolemo:z.inf_age	0.448	0.287	0.606
rolemo:z.mean.inf.age	-0.332	-0.455	-0.206
infant@role.code	0.233	0	0.281
infant.1@z.inf_age	0	0	0
infant.2@urgency	0	0	0
infant.3@int.r.ia	0.048	0	0.161
infant.4@(Intercept)	0	0	0
mother@(Intercept)	0	0	0.069

**Visual gesture**	orig	min	max
(Intercept)	-3.047	-3.498	-2.941
rolemo	2.272	2.12	2.549
z.inf_age	0.067	-0.302	0.409
z.mean.inf.age	-0.035	-0.78	0.103
urgency	0.481	0.416	0.549
inf_sexM	-0.109	-0.283	0.111
group.L	0.107	0.047	0.198
group.Q	0.124	0.066	0.177
z.mot_exp	0.1	0.03	0.254
siteT	0.039	-0.279	0.274
rolemo:z.inf_age	-0.075	-0.353	0.282
rolemo:z.mean.inf.age	0.22	0.121	0.807
infant@role.code	0.156	0	0.178
infant.1@z.inf_age	0.223	0	0.247
infant.2@urgency	0	0	0.101
infant.3@int.r.ia	0	0	0.204
infant.4@(Intercept)	0	0	0
mother@(Intercept)	0	0	0

**Initiator**	orig	min	max
(Intercept)	-1.804	-2.282	-1.074
z.inf_age	0.546	0.422	0.676
z.mean.inf.age	0.792	0.639	1.214
urgency	0.022	-0.136	0.194
inf_sexM	0.053	-0.324	0.302
groupfemales	0.803	0.539	1.18
groupmixed	0.637	0.347	0.96
z.mot_exp	0.688	0.534	0.928
siteT	0.351	-0.088	0.605
dyad@z.inf_age	0.613	0.333	0.718
dyad.1@urgency	0	0	0
dyad.2@(Intercept)	0	0	0.151
